# Sphingosine is involved in PAPTP-induced death of pancreas cancer cells by interfering with mitochondrial functions

**DOI:** 10.1007/s00109-024-02456-2

**Published:** 2024-05-23

**Authors:** Sameer H. Patel, Gregory C. Wilson, Yuqing Wu, Simone Keitsch, Barbara Wilker, Andrea Mattarei, Syed A. Ahmad, Ildiko Szabo, Erich Gulbins

**Affiliations:** 1 Department of Surgery, University of Cincinnati College of Medicine, Cincinnati, OH USA; 2 Institute of Molecular Biology, University Hospital Essen, University of Duisburg-Essen, Hufelandstrasse 55, 45122 Essen, Germany; 3 Department of Pharmaceutical and Pharmacological Sciences, University of Padua, Padua, Italy; 4 Department of Biology, CNR Institute of Neurosciences, University of Padua, Padua, Italy

**Keywords:** Pancreas cancer, Sphingosine, Cytochrome C, Kv1.3, Mitochondria

## Abstract

**Abstract:**

Pancreas ductal adenocarcinoma belongs to the most common cancers, but also to the tumors with the poorest prognosis. Here, we pharmacologically targeted a mitochondrial potassium channel, namely mitochondrial Kv1.3, and investigated the role of sphingolipids and mutated Kirsten Rat Sarcoma Virus (KRAS) in Kv1.3-mediated cell death. We demonstrate that inhibition of Kv1.3 using the Kv1.3-inhibitor PAPTP results in an increase of sphingosine and superoxide in membranes and/or membranes associated with mitochondria, which is enhanced by KRAS mutation. The effect of PAPTP on sphingosine and mitochondrial superoxide formation as well as cell death is prevented by sh-RNA-mediated downregulation of Kv1.3. Induction of sphingosine in human pancreas cancer cells by PAPTP is mediated by activation of sphingosine-1-phosphate phosphatase and prevented by an inhibitor of sphingosine-1-phosphate phosphatase. A rapid depolarization of isolated mitochondria is triggered by binding of sphingosine to cardiolipin, which is neutralized by addition of exogenous cardiolipin. The significance of these findings is indicated by treatment of mutated KRAS-harboring metastasized pancreas cancer with PAPTP in combination with ABC294640, a blocker of sphingosine kinases. This treatment results in increased formation of sphingosine and death of pancreas cancer cells in vitro and, most importantly, prolongs in vivo survival of mice challenged with metastatic pancreas cancer.

**Key messages:**

- Pancreatic ductal adenocarcinoma (PDAC) is a common tumor with poor prognosis.
- The mitochondrial Kv1.3 ion channel blocker induced mitochondrial sphingosine.
- Sphingosine binds to cardiolipin thereby mediating mitochondrial depolarization.
- Sphingosine is formed by a PAPTP-mediated activation of S1P-Phosphatase.
- Inhibition of sphingosine-consumption amplifies PAPTP effects on PDAC in vivo.

## Introduction

Pancreatic ductal adenocarcinoma (PDAC) is the third leading cause of cancer-related death in the United States. Unfortunately, the majority of patients with pancreatic cancer are metastatic at the time of diagnosis or already developed early micrometastatic disease with seemingly localized disease. This is seen with systemic recurrences despite treatment with surgery alone. The cornerstone of treatment to reduce recurrences remains systemic chemotherapy. Despite incremental improvements in treatment outcomes, results remain abysmal with 5-year survival just recently surpassing 10% [1, 2]. Attempts to improve treatment efficacy, for instance by introducing check-point inhibitors to trigger an immune response against the tumor, have largely been studied with negative results [1, 2]. These failures highlight our lack of knowledge and understanding of the molecular underpinnings of the aggressive nature of this malignancy. Here, we focused on the role of a mitochondrial ion channel as a potential target for the treatment of PDAC. 

Ion channels are transmembrane proteins that form aqueous pores driving ions through the plasma membrane in favor of an electrochemical gradient. We have previously demonstrated that Kv1.3 is not only expressed on the cell membrane, but also in the inner mitochondrial membrane. The voltage-gated Kv1.3 channel of the Kv1 subfamily is mostly expressed in immune cells, including T and B lymphocytes, macrophages, neutrophils, microglia, and dendritic cells and in neurons [3, 4]. However, we and others have previously shown the expression of Kv1.3 in numerous types of cancers [5–7]. The mitochondrial Kv1.3 channel has been shown to be very important for the induction of cell death in a variety of cells, including cancer cells [8, 9].

In detail, inhibition of mitochondrial Kv1.3 results in changes in mitochondrial membrane potential, formation of reactive oxygen species (ROS), opening of the permeability transition pore (PTP) [10], release of cytochrome c, and cell death [8, 9, 11, 12]. All of these events are abrogated in Kv1.3-deficient cells. The formation of ROS upon inhibition of mitochondrial Kv1.3 was shown to be a crucial step in the induction of cell death [9]. We developed two novel mitochondria-targeted specific inhibitors of Kv1.3, i.e., PAPTP and PCARBTP, and demonstrated that the basal level of ROS production and expression levels of mitochondrial Kv1.3 determine whether these inhibitors kill cells or not [9]. The new drugs allowed us to target exclusively malignant tumor cells, because their basal levels of ROS and Kv1.3 expression level are higher than those of non-malignant cells [13, 14]. However, many details of the mechanisms of how Kv1.3 inhibitors induce cell death are still unknown.

Here, we tested a role of sphingolipids in killing of pancreas cancer cells by inhibitors of mitochondrial Kv1.3. and established their importance in the context of cancer therapy. Sphingolipids are a class of lipids that share an amino alcohol (sphingoid base) backbone. Ceramides are a subfamily of sphingolipids in which the amino group on sphingosine is linked to a fatty acid [15]. We have previously shown that ceramide alters the organization and the distribution of receptors and associated signalling molecules within membranes, and this activity allows the generation of intracellular signals [16]. Thus, ceramide may act via a change in membrane biophysics that results in the formation of ceramide-enriched membrane domains and the reorganization of receptors and signalling molecules within these domains [16], but it may also act by direct interaction with certain proteins [17–22]. Sphingosine may regulate certain protein kinase C isoforms [23], but at present it is largely unknown how sphingosine acts on and in cells. It was shown that under stress, ceramide synthase 1 traffics from the endoplasmic reticulum to the cytoplasmic leaflet of the outer mitochondrial membrane and generates C18 ceramide [22]. Mitochondrial C18 ceramide in the cytosolic leaflet of the outer mitochondrial membrane binds to LC3-II and thereby mediates mitophagy [22]. At present, the functions of mitochondrial sphingosine are completely unknown; however, general consensus is that sphingosine is pro-apoptotic, while sphingosine-1 phosphate (S1P) is mitogenic and anti-apoptotic [24].

Here, we demonstrate that inhibition of mitochondrial Kv1.3 using one of the inhibitors we recently developed, i.e., PAPTP, results in the formation of mitochondrial/mitochondria-associated sphingosine. Sphingosine binds to cardiolipin in mitochondrial membranes and induces a rapid depolarization of mitochondria, which may trigger cell death. In addition, we explored the role of sphingosine in KRAS modulation, since sphingomyelin metabolism has been proposed to act as a KRAS regulator [25]. Activating point mutations in RAS genes are associated with approximately 30% of all human cancers. In particular, mutant KRAS is the most oncogenic and frequent, accounting for 85% of all mutated RAS proteins reported, and is predominantly found in PDAC. Interestingly, the mitochondria-targeted anti-oxidant MitoQ has been shown to decrease KRAS-caused formation of PDAC in vivo [26], suggesting that mitochondria are the primary source of KRASG12D-induced ROS. This is in agreement with a translocation of mutant KRAS to mitochondria [27]. We hypothesize that oncogenic KRAS alters the physiology/metabolism of mitochondria resulting in increased sensitivity to mitoKv1.3 inhibition and serves as a paradigm for alterations in tumor cells or pharmacologically induced manipulations that result in increased mitochondrial ROS and sensitize cancer cells to these drugs. 

## Methods

### Mice and in vivo studies

## Ethics approval

All animal experiments were approved by the University of Cincinnati Ethics Committee and the Institutional Animal Care and Use Committee.

All experiments were performed according to the FELASA regulations and we also followed the ARRIVE guidelines.

Eight- to 12-week-old, wild-type C57BL/6 J mice were employed. Pancreas cancer was established in the peritoneum by intraperitoneal injection of 50,000 KPC cells resuspended in PBS. Mice were treated i.v. 6 times starting at day 3 and then every 2nd day with 4 nmol/g PAPTP + 50 mg/kg ABC294640. PAPTP was injected i.v. in a total volume of 125 µL DMSO and 0.9% NaCl (1:4, v/v); ABC294640 was injected i.p. Controls were injected with the solvent only. Survival was determined over 65 days.

Tumor-injected mice were randomly divided into experimental groups.

### Cell culture and treatment

Human PDAC cell lines BxPc3, Panc-1, and MIA Paca-2 were obtained from the pHionic consortium. KPC tumor cells were originally generated from *KRAS*^*LSL−G12D/*+^, *Trp53*^*loxP/*+^, and *Pdx1-Cre* (KPC) mice in C57BL/6 mice background (Jackson Laboratories). Rodent pathogen status of the tumor cell lines was originally verified by Charles River Laboratory diagnosis services.

All cells were cultured in modified Eagle medium (MEM) supplemented with 10 mM HEPES (pH 7.4; Carl Roth GmbH, Karlsruhe, Germany), 2 mM L-glutamine, 1 mM sodium pyruvate, 100 µM nonessential amino acids, 100 U/mL penicillin, 100 µg/mL streptomycin, and 10% fetal calf serum (MEM/K10).

If indicated, cells were treated with 10 µM XY-14 (Echelon, #L9218) for 30 min prior to the addition of PAPTP.

### Transfections

Panc-1 and MIA Paca-2 cells expressing wild-type or oncogenic RAS were transfected with shRNA targeting KRAS or sphingosine kinase 2 (all from Santa Cruz Inc., #sc-35731-SH for KRAS, sc-39225-SH for sphingosine kinase 2). Cells were stably transfected by electroporation.

Kv1.3 was downregulated by transient transfection using shRNA targeting Kv1.3 (Santa Cruz Inc., #sc-42712-SH) using a BTX electroporator. Cells were employed 48 h after transient transfection.

For transfections, cells were trypsinized and washed 3 times in HEPES/saline (H/S; 132 mM NaCl, 20 mM HEPES [pH 7.4], 5 mM KCl, 1 mM CaCl_2_, 0.7 mM MgCl_2_, 0.8 mM MgSO_4_). Cells were then resuspended in H/S at 20 × 10^6^ cells/400 µL, aliquoted into electroporation cuvettes, the plasmids were added, and samples were incubated on ice for 15 min and electroporated at 500 V with 5 pulses each for 1 ms. Cells were incubated for 15 min on ice and finally resuspended in MEM/K10. Transfected cells were then selected by incubation with 3.5 µg/mL puromycin for 3–4 weeks. We used bulk cultures in the present experiments to avoid any clonal artifacts.

### Reactive oxygen species (ROS)

Cells were grown in MEM/K10 as above, removed from the plates using cell dissociation buffer (ThermoFisher, #13,151,014), washed in PBS, incubated with 2.5 µM mitoSOX Red (ThermoFisher, # M36008) for 20 min at 37 °C, and stimulated with 0.25 or 1 µM PAPTP or left untreated. Cells were then analyzed by flow cytometry employing a FACSCalibur.

### Cell death

Cells were treated as indicated, trypsinized, washed in H/S, resuspended in a Ca^2+^-containing staining buffer as provided by the vendor, and stained for 15 min with FITC-Annexin V (1:1000, Roche). Samples were analyzed by flow cytometry on a FACSCalibur. Positive controls were permeabilized for 5 min with 0.1% Triton X-100 at room temperature before incubation with FITC-Annexin V.

### Protein measurements

Protein concentrations were measured employing the BioRad Protein Assay Dye (cat. no. #500,006) and served to normalize the samples.

### Sphingosine measurements

Cells were grown in 24-well plates overnight, washed, and stimulated with 1 µM PAPTP for 30 min in H/S or left untreated, the medium was removed, and cells were washed once with cold H/S and shock-frozen in liquid nitrogen. Cells were then lysed in 250 µL 0.5% NP40 in H_2_O and 200 µL was extracted in 600 µL CHCl_3_:CH_3_OH:1N HCl (100:100:1, v/v/v). Phases were separated, and an aliquot of the lower phase was dried in a Speedvac and resuspended in 20 µL of a detergent solution (7.5% [w/v] n-octyl glucopyranoside, 5 mM cardiolipin in 1 mM diethylenetriaminepentaacetic acid [DTPA]). The samples were sonicated in a bath sonicator for 10 min and the kinase reaction was performed by the addition of 80 µL of 0.001 units sphingosine kinase in 50 mM HEPES (pH 7.4), 250 mM NaCl, 30 mM MgCl_2_ 1 mM ATP, and 10 µCi [^32^P]γATP. The kinase reaction was performed for 30 min and terminated by extraction of the samples in 20 µL 1N HCl followed by the addition of 800 µL CHCl_3_:CH_3_OH:1N HCl (100:200:1, v/v/v), and 240 µL each of CHCl_3_ and 2 M KCl. Samples were vortexed between additions. Phases were separated, and the lower phase was collected, dried, dissolved in 20 µL CHCl_3_:CH_3_OH (1:1, v/v), and separated on Silica G60 TLC plates with CHCl_3_:CH_3_OH:acetic acid:H_2_O (90:90:15:5, v/v/v/v) as developing solvent. The TLC plates were analyzed with a phosphorimager. Sphingosine levels were determined with a standard curve of C18-sphingosine.

### Ceramide measurements

Cells were treated and extracted as described for sphingosine. The dried samples were resuspended in 20 µL of a detergent solution (7.5% [w/v] n-octyl glucopyranoside and 5 mM cardiolipin in 1 mM diethylenetriamine-pentaacetic acid [DTPA]), and micelles were obtained by bath sonication for 10 min. The kinase reaction was initiated by the addition of 70 µL of a reaction mixture containing 10 µL diacylglycerol (DAG) kinase (GE Healthcare Europe, Munich, Germany), 0.1 M imidazole/HCl (pH 6.6), 0.2 mM DTPA, 70 mM NaCl, 17 mM MgCl_2_, 1.4 mM ethylene glycol tetraacetic acid, 2 mM dithiothreitol, 1 µM adenosine triphosphate (ATP), and 5 µCi [^32^P]γATP. The kinase reaction was performed for 30 min at room temperature under shaking at 300 rpm, terminated by the addition of 1 mL CHCl_3_:CH_3_OH:1N HCl (100:100:1, v/v/v), 170 µL buffered saline solution (135 mM NaCl, 1.5 mM CaCl_2_, 0.5 mM MgCl_2_, 5.6 mM glucose, and 10 mM HEPES; pH 7.2), and 30 µL of a 100 mM ethylenediaminetetraacetic acid (EDTA) solution. The samples were vortexed, phases were separated, and the lower phase was collected, dried, dissolved in 20 µL CHCl_3_:CH_3_OH (1/1, v/v), separated on Silica G60 thin-layer chromatography (TLC) plates using chloroform/acetone/methanol/acetic acid/H_2_O (50:20:15:10:5, v/v/v/v/v) as solvent, and developed employing a Fuji phosphorimager. Ceramide levels were determined by comparison with a standard curve; C16 to C24 ceramides were used as substrates.

### Sphingosine-1-phosphate-phosphatase activity

Cells were stimulated with 1 µM PAPTP for 30 min or left untreated, and mitochondria were prepared as above and incubated with 10 µM [^3^H]S1P complexed with fatty acid–free BSA for 30 min at 37 °C. The samples were organically extracted as described above for sphingosine measurements, separated by TLC, and consumption of S1P to sphingosine was determined.

### Sphingosine kinase activity

Cells were stimulated with 1 µM PAPTP for 30 min or left untreated; mitochondria were prepared as above and resuspended after purification in a buffer consisting of 50 mM HEPES, pH 7.4, 150 mM NaCl, 30 mM MgCl_2_ in the presence of 500 pmol sphingosine (i.e., excess substrate), 10 µM ATP, and 10 µCi[^32^P]γATP. The kinase reaction was allowed to proceed for 60 min at 30 °C and stopped by the addition of 20 µL 1N HCl followed by the addition of 800 µL CHCl_3_:CH_3_OH:1N HCl (100:200:1, v/v/v), and 240 µL each of CHCl_3_ and 2 M KCl. Samples were then processed as described above for sphingosine measurements.

### Measurement of cardiolipin binding to sphingosine

Cells were treated with 1 µM PAPTP for 30 min and lysed in 125 mM NaCl, 25 mM Tris HCl (pH 7.4), 10 mM EDTA, 10 mM sodium pyrophosphate, 2% Nonidet P40, and 10 µg/mL aprotinin and leupeptin for 10 min on ice. Sphingosine was immunoprecipitated from the lysates using a monoclonal anti-sphingosine antibody (clone NHSPH, Alfresa Pharma Corporation, Japan) for 60 min at 4 °C. Immunocomplexes were immobilized by incubation with protein A/G-coupled agarose for 45 min at 4 °C. The precipitates were extensively washed 6 times in H/S and extracted in CHCl_3_/CH_3_OH/1N HCl (100:100:1, v/v/v). Samples were dried and then resuspended in 10 µM of a cardiolipin probe (Abcam, ab241036), a fluorescent probe for the detection and quantification of cardiolipin. The samples were incubated for 5 min, 250 rpm shaking and the fluorescence was measured using a fluorescence reader. Cardiolipin concentration was determined using a standard curve of cardiolipin.

### Incubation of isolated mitochondria with sphingosine and measurement of mitochondrial membrane potential in vitro

Mitochondria were isolated as above, incubated with 2.5 nM TMRM (ThermoFisher M20036) for 5 min at 37 °C and incubated with 100 nM sphingosine (Avanti Polar Lipids, #860,490) or 100 nM PAPTP. If indicated, we added 10 µM Cardiolipin (Merck, #C0563). Samples were analyzed by flow cytometry using a FACSCalibur. The results were analyzed for the mean fluorescence using the BD software.

### Flow cytometry

Cells were treated with sphingosine, resuspended in buffer A consisting of 0.3 M sucrose, 10 mM TES (pH 7.4), and 0.5 mM EGTA, and incubated for 30 min on ice. Cells were then pottered in a tight glass potter with 40 strokes, centrifuged at 600 × *g* for 5 min at 4 °C, and the supernatants were collected. The supernatants were centrifuged at 6000 × *g* for 10 min at 4 °C, the pellets were resuspended in buffer B consisting of 50 mM Pipes-KOH (pH 7.4), 50 mM KCl, 2 mM MgCl_2_, 2 mM EGTA, 10 µg/mL A/L, 2 mM ATP, 10 mM phosphocreatine, 5 mM succinate, and 50 µg/mL creatine kinase, and samples were stained for 30 min with anti-TIM23 (1:250 dilution; BD, #611,222) and anti-sphingosine antibodies (1:1000 dilution, Alfresa, clone NHSPH) or anti-Kv1.3 antibodies (1:250, Alamone, #APC-101), respectively. Samples were washed and stained with Cy3-coupled donkey anti-mouse IgG F(ab)_2_ fragments (1:500 dilution; Jackson Immunoresearch, #715–166-150) for the mouse anti-TIM23 IgG + Alexa Fluor 647-coupled donkey anti-mouse IgM F(ab)_2_ fragments (1:500 dilution; Jackson Immunoresearch, #715–606-020) for the IgM anti-sphingosine or APC-coupled donkey anti-rabbit IgG F(ab)_2_ fragments (1:500 dilution; Jackson Immunoresearch, #711–136-152) for the rabbit anti-Kv1.3 IgG. Samples were washed again and analyzed by flow cytometry using a FACSCalibur employing FL2 vs. FL4. The mean fluorescence was analyzed using BD software.

### Statistics

Data are expressed as arithmetic means ± SD. For the comparison of continuous variables from independent groups with one variable, we used one-way ANOVA followed by post hoc Tukey test for all pairwise comparisons, applying the Bonferroni correction for multiple testing. The *P* values for the pairwise comparisons were calculated after the Bonferroni correction. All values were tested for normal distribution using the Kolmogorov–Smirnov test. Statistical significance was set at a *P* value of 0.05 or lower (two-tailed). Outliers were not removed and all data are provided. The sample size planning was based on the results of two-sided Wilcoxon-Mann–Whitney tests (free software: G*Power, Version 3.1.7, University of Duesseldorf, Germany). Investigators were blinded to the samples in microscopic studies and to animal identity. Animals were randomly assigned to cages by a technician who was not involved in the experiments; thus, the mice were purely randomly assigned for every experiment. Cages were then randomly assigned to the various experimental groups.

## Results

### KRAS influences PAPTP-induced death of pancreas ductal adenocarcinoma cells

**Fig. 1:**
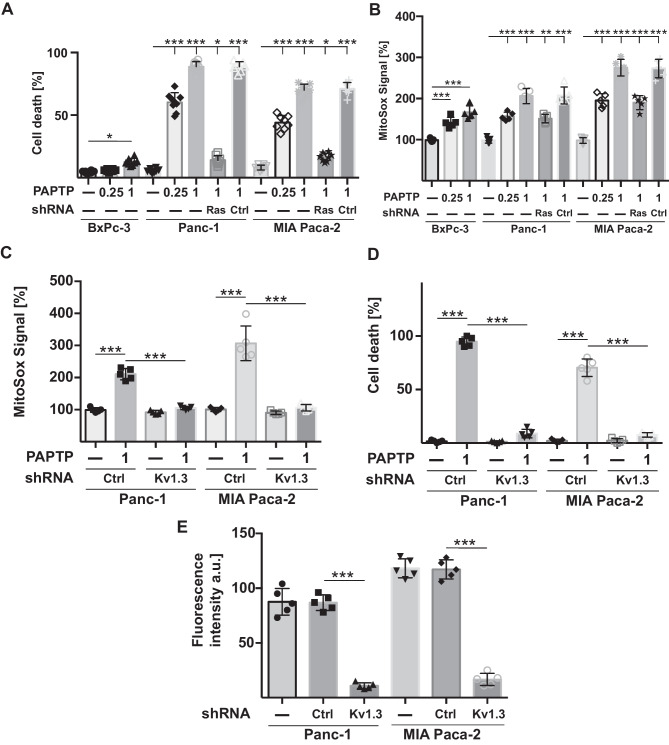
Oncogenic RAS sensitizes pancreas cancer to PAPTP-induced cell death. **A** PDAC cells expressing wild-type or oncogenic RAS or cells that were transfected with shRNA targeting RAS or control shRNA were treated for 48 h with PAPTP (0.25 or 1 µM) and cell death was determined by FITC-Annexin-V staining and flow cytometry analysis. Displayed are the mean ± SD, *n* = 8, ***p < 0.001, ANOVA and post hoc Tukey test. **B** Reactive oxygen species were determined by loading the cells with mitoSox and flow cytometry analysis upon treatment with PAPTP. Expression of oncogenic Ras increased mitochondrial ROS, and this sensitization was reversed by shRNA-mediated suppression of RAS expression in pancreas carcinoma cells (**B**). PAPTP induces a release of ROS. Shown are the mean ± SD, *n* = 5, ***p < 0.001, ANOVA and post hoc Tukey test. **C–E** Kv1.3 was downregulated in Panc-1 or MIA Paca-2 cells by transfection of siRNA targeting Kv1.3. Controls (Ctrl) were transfected with irrelevant siRNA constructs. Si-RNA-mediated downregulation of Kv1.3 prevented PAPTP-induced cell death (**C**) and mitochondrial superoxide release (**D**). **E** Control flow cytometry studies confirm the downregulation of Kv1.3 in mitochondria upon siRNA transfection. Given are the mean ± SD, *n* = 5, ***p < 0.001, ANOVA and post hoc Tukey test

To define mechanisms that determine the mitochondrial effects of PAPTP, we tested whether oncogenic KRAS, which drives tumorigenesis in pancreas cancer, influences the effects of PAPTP on tumor cells. We treated human BxPc-3 pancreas cancer cells, which express wild-type (wt) RAS, and Panc-1 and MiaPaCa-2 cells, which both express oncogenic Ras (Gly12Asp-RAS, Gly12Cys-RAS) [28], with 250 nM and 1 µM PAPTP for 48 h. To test whether oncogenic KRAS alters death by PAPTP, we down-modulated KRAS in Panc-1 and MiaPaCa-2 cells by transfection of shRNA targeting KRAS. Downregulation was confirmed by western blotting (not shown). The results show that 75–90% of the pancreas cancer cells expressing oncogenic KRAS died after treatment with PAPTP, while cells expressing wild-type KRAS or cells transfected with shRNA targeting KRAS were less sensitive to PAPTP (Fig. 1A).

Expression of oncogenic KRAS resulted in an increase of mitochondrial ROS in accordance with, e.g., [26]. The increased release of ROS in cells expressing oncogenic KRAS was reduced by shRNA-mediated suppression of KRAS expression in pancreas carcinoma cells (Fig. 1B). PAPTP treatment drastically enhanced the release of ROS, which was much higher in PDAC-expressing oncogenic KRAS than in those lacking oncogenic RAS (Fig. 1B). We hypothesize that oncogenic KRAS mediates increased sensitivity to PAPTP treatment that results in increased mitochondrial ROS and sensitize cancer cells.

Downregulation of Kv1.3 in Panc-1 or MIA Paca-2 cells, respectively, prevented the effects of PAPTP on mitochondrial superoxide release and cell death, confirming Kv1.3 as target for PAPTP (Fig. 1C,D). It is important to note that a downregulation of Kv1.3, which allows the cells to compensate and maintain the mitochondrial membrane potential, differs from the acute inhibition of the channel by an inhibitor resulting in membrane potential changes as a signal as previously described [9, 11, 12]. Downregulation of Kv1.3 in mitochondria was confirmed by staining isolated mitochondria from transfected cells with APC-anti-Kv1.3 and Cy3-anti-TIM23 followed by flow cytometry analysis and determination of the mean fluorescence signal for APC (Fig. 1E) in the Cy3-positive population.

### PAPTP induces sphingosine in mitochondria

**Fig. 2:**
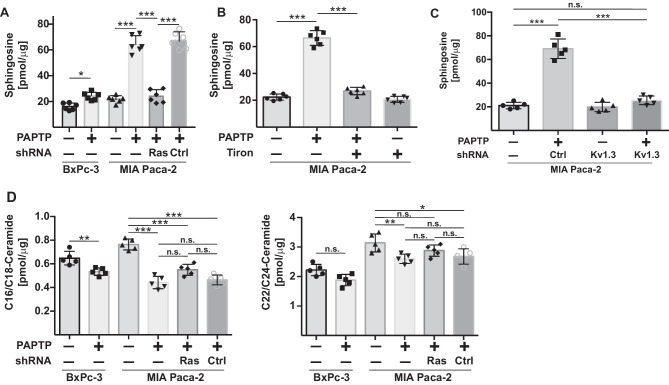
PAPTP induces the formation of sphingosine in mitochondria/mitochondria-associated membranes of pancreas cancer cells expressing oncogenic RAS, while cells lacking oncogenic RAS release much less sphingosine. **A**, **B** MiaPaca-2 PDAC cells expressing oncogenic RAS were transiently transfected with shRNA targeting RAS, control shRNA, or left untransfected. BxPc-3 lacking oncogenic RAS were used as controls. Cells were stimulated with 250 nM PAPTP in the presence or absence of the 10 µM anti-oxidant Tiron for 30 min and lysed, mitochondria were isolated and organically extracted, and sphingosine was quantified using kinase assays. **C** Downregulation of Kv1.3 in MIA Paca-2 cells using siRNA transfections prevented the formation of sphingosine by PAPTP. Shown is the quantitative analysis of the mean fluorescence of the sphingosine signal (FL4 channel) in the Cy3-positive population (FL2 channel) that represents mitochondria. Displayed are the mean ± SD, *n* = 5 or 6; *p < 0.001 ANOVA and post hoc Tukey’s multiple comparison test. **D** Ceramide was determined in cells treated with PAPTP or left untreated by a ceramide kinase assay. Shown are the mean ± SD, *n* = 5; *p < 0.001 ANOVA and post hoc Tukey’s multiple comparison test

To identify targets of PAPTP-induced ROS, we tested the effects of PAPTP on sphingolipid metabolism, since the sphingolipid metabolism is redox-sensitive [29, 30]; we tested whether PAPTP induces the formation of intracellular ceramide and sphingosine. Our data indicate a marked increase of mitochondria-associated sphingosine concentrations upon treatment of pancreas cancer cells with PAPTP (Fig. 2A–C). We also observed a decrease of ceramide upon treatment with PAPTP (Fig. 2D). The formation of sphingosine was greatly enhanced in cells expressing oncogenic RAS, such as MIA Paca-2 cells, compared to cells not expressing oncogenic RAS, such as BxPc3 (Fig. 2A). Downregulation of KRAS by shRNA also decreased the formation of sphingosine in pancreas cancer cells (Fig. 2A). Furthermore, neutralization of ROS by incubating the cells with the anti-oxidant Tiron also reduced the release of sphingosine from pancreas cancer cells upon treatment with PAPTP (Fig. 2B). Si-RNA-mediated downregulation of Kv1.3 in MIA Paca-2 cells prevented the induction of sphingosine by PAPTP (Fig. 2C).

**Fig. 3:**
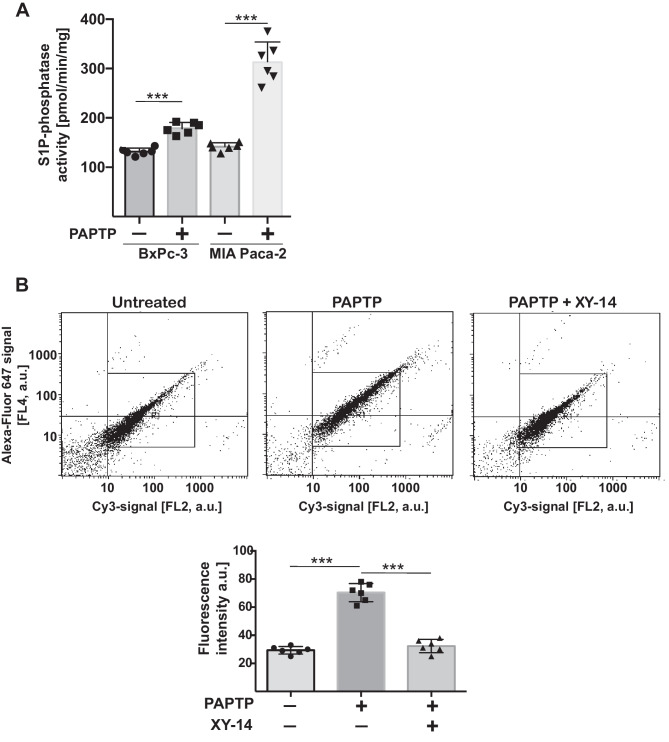
PAPTP induces the activity of sphingosine-1 phosphate-phosphatase. **A** Cells were disrupted and mitochondria isolated and incubated with 10 µM [^3^H]S1P complexed with fatty acid–free BSA for 30 min at 37 °C, organically extracted, and separated by TLC, and consumption of S1P to sphingosine was determined. **B** MiaPaca-2 PDAC cells were treated with PAPTP for 30 min in the presence or absence of the S1P-phosphatase inhibitor XY-14, swollen, and pottered, and mitochondria were isolated. The samples were then stained with Cy3-coupled anti-TIM23 antibodies and Alexa Fluor 647-coupled anti-sphingosine antibodies and analyzed by flow cytometry. Shown are a typical flow cytometry result and the quantitative analysis of the mean fluorescence of the sphingosine signal (FL4 channel) in the Cy3-positive population (FL2 channel) that represents mitochondria. This population is indicated by the square. Shown are the mean ± SD, *n* = 6, or representative stainings from 6 independent experiments; ***p < 0.001 ANOVA and post hoc Tukey test

To identify mechanisms of how PAPTP induces the formation of sphingosine, we tested the activity of the sphingosine 1-phosphate-(S1P-)phosphatase in mitochondria/mitochondria-associated membranes of oncogenic KRAS-expressing or lacking cells (Fig. 3A). Sphingosine can be generated from sphingosine 1-phosphate (S1P) by the activity of a S1P-phosphatase. The results show that PAPTP induces an activation of S1P-phosphatase, which is enhanced in cells expressing oncogenic KRAS, compared to pancreas cancer cells lacking oncogenic KRAS (Fig. 3A). We did not observe a change of sphingosine kinase activity upon treatment with PAPTP in pancreas cancer cells (not shown).

Next, to confirm the formation of sphingosine in mitochondria and the role of S1P-phosphateses, we treated MIA Paca-2 cells with PAPTP in the presence or absence of the S1P inhibitor XY-14 [31]. Cells were then disintegrated, mitochondria isolated and stained with Cy3-anti-TIM23 and Alexa Fluor 647-anti-sphingosine. Samples were then analyzed by flow cytometry and the fluorescence signal intensity of the TIM23 positive population was determined. The results indicate a formation of sphingosine in mitochondria upon treatment with PAPTP, which was abrogated by treatment with the S1P-inhibitor XY-14 (Fig. 3B).

### Sphingosine binds to cardiolipin in mitochondrial membranes

**Fig. 4:**
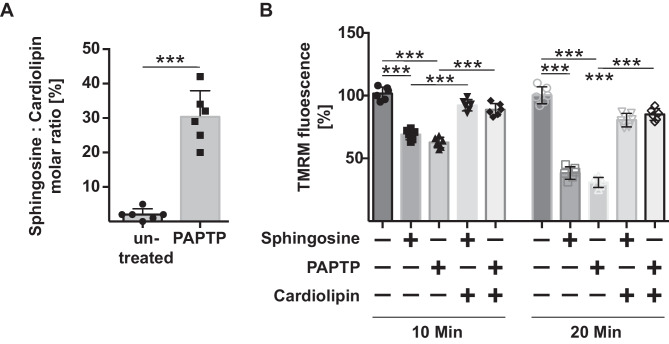
Sphingosine binds to cardiolipin upon treatment of cells with PAPTP. **A** MiaPaca-2 cells were treated with 1 µM PAPTP for 30 min and lysed and sphingosine was immunoprecipitated from the lysates to detect associating cardiolipin. Shown is the mean ± SD of the ratio of cardiolipin, *n* = 4, ***p < 0.001 ANOVA and post hoc test. **B** Isolated mitochondria were loaded with TMRM and incubated with 100 nM sphingosine or 100 nM PAPTP, and the mitochondrial membrane potential was determined by flow cytometry 10 min and 20 min after the addition of compounds. Addition of cardiolipin (10 µM) greatly reduced the effects of sphingosine and PAPTP on mitochondrial membrane potential determined after 10 and 20 min using the same laser settings for all samples. Shown is the mean ± SD of the fluorescence determined by flow cytometry, *n* = 6, ***p < 0.001 ANOVA and post hoc test

We have previously shown that sphingosine binds to cardiolipin and thereby mediates rapid permeabilization of bacterial membranes and bacterial cell death [32]. Bacterial cell death was mediated by a massive, sphingosine-mediated clustering of cardiolipin resulting in the generation of very rigid membrane domains and finally in membrane leakiness, depolarization, loss of ATP, and bacterial cell death [32]. The endosymbiont hypothesis indicates that mitochondria and bacteria are similar, and in fact in mammalian cells only the inner mitochondrial membrane contains cardiolipin. Here, we show that PAPTP induced binding of sphingosine to cardiolipin in mitochondria after stimulation (Fig. 4A). Sphingosine and PAPTP induced rapid depolarization of isolated mitochondria (Fig. 4B), which was prevented by co-incubation of the mitochondria with exogenous cardiolipin (Fig. 4B).

### Pharmacological or genetic manipulation of sphingosine formation determines PAPTP-induced tumor cell death in vitro and in vivo

**Fig. 5:**
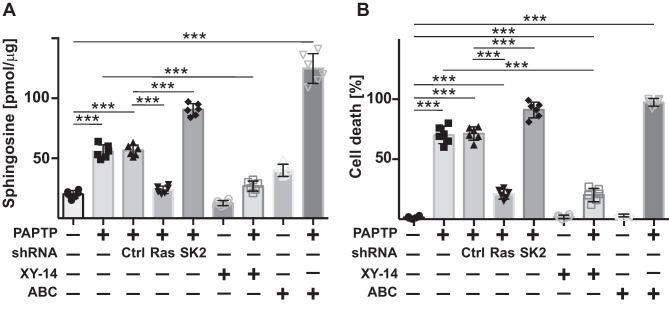
Pharmacological inhibition of S1P-phosphatase prevents the formation of sphingosine in and cell death of pancreas cancer cells. MIA PaCa-2 cells were treated with 10 µM S1P-inhibitor XY-14 or 10 µM of the sphingosine kinase 2 (SK2) inhibitor ABC294640 or transfected with siRNA targeting RAS or sphingosine kinase 2. Untransfected or control vector-transfected cells (Ctrl) served as controls. Cells were treated with 1 µM PAPTP for 30 min to determine sphingosine **A** or 48 h to measure cell death **B**. Cells were lysed, mitochondria isolated, and sphingosine quantified by kinase assays; cell death was measured by FITC-Annexin V staining and flow cytometry. Given are the mean ± SD, *n* = 6; *p < 0.001 ANOVA and post hoc Tukey’s multiple comparison test

If PAPTP-induced sphingosine is involved in cell death, inhibition of S1P-phosphatase should prevent cell death induced by PAPTP. We therefore treated BxPc-3 PDAC (wt RAS) and MiaPaCa-2 cells (Gly12Cys KRAS) with 250 nM PAPTP in the presence or absence of 10 µM of the S1P phosphatase inhibitor XY-14. The results show that PAPTP-induced sphingosine formation and death of pancreas cancer cells were greatly reduced by S1P-phosphatase inhibition (Fig. 5A).

Although PAPTP does not regulate sphingosine kinase 2, sphingosine kinase 2 consumes sphingosine by its constitutive activity and counteracts S1P-phosphatase. Thus, we also inhibited sphingosine kinase 2 using ABC294640 [33]. The results show that PAPTP-induced formation of sphingosine (Fig. 5A) and cell death (Fig. 5B) of pancreas cancer cells were increased upon pharmacological inhibition of sphingosine kinase 2.

**Fig. 6:**
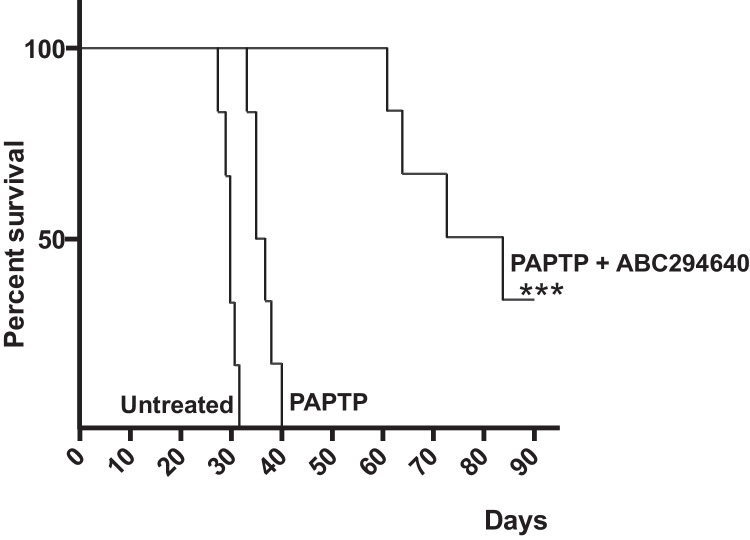
The combination of PAPTP and carmofur allows long-term survival of mice with metastasized pancreas cancer. Metastasized pancreas cancer was established in the peritoneum by injection of 50,000 KPC cells; mice were treated i.v. 6 times starting at day 3 with 4 nmol/g PAPTP + every 2nd day with one i.p. injection/day of 50 mg/kg ABC294640. Controls were injected with solvent. Survival was determined over 60 days. Six mice were investigated per group. ***p < 0.001 compared to the survival of untreated or PAPTP-treated mice

To test the significance of these findings, we established a metastatic pancreas cancer model by intraperitoneal injection of 50,000 PDAC cells. Treatment with PAPTP and ABC294640 markedly delayed tumor growth in vivo (Fig. 6), while ABC294640 alone had no effect on pancreas cancer growth (not shown).

## Discussion

**Fig. 7:**
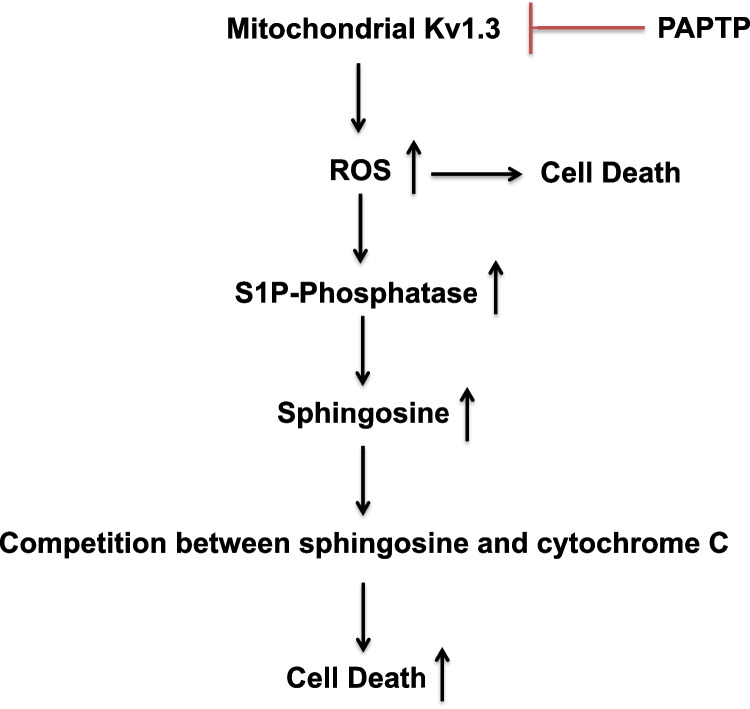
Scheme of the proposed PAPTP-induced mechanisms

The present data suggest that ROS-triggered sphingosine significantly contributes to PAPTP-induced cell death. The levels of sphingosine are controlled (1) by hydrolysis of sphingosine-1-phosphate by a S1P-phosphatase associated with mitochondrial fractions and (2) by sphingosine kinases, which are not affected by PAPTP, but constitutively consume sphingosine. It is certainly possible that ceramidases by their activity also contribute to mitochondrial sphingosine, but the exact definition of the function and regulation of these enzymes in mitochondria seems to be beyond the present manuscript. Our data suggest that sphingosine binds to cardiolipin and induces rapid depolarization of isolated mitochondria, which is neutralized by the addition of exogenous cardiolipin (Fig. 7). Thus, a change of mitochondrial membrane biophysics, in particular fluidity, might be at least in part a mechanism how sphingosine mediates cell death.

We have previously shown that sphingosine binds via the protonated NH_2_ group to cardiolipin in bacterial and model membranes and thereby mediates a marked change in the fluidity and also the permeability of bacterial and model membranes [32]. Here, we suggest that sphingosine synthesized in mitochondria or associated mitochondrial membranes has a similar effect and induces rapid induction of mitochondrial membrane potential loss, possibly thereby mediating cell death. This might be one additional mechanism how PAPTP-mediated inhibition of Kv1.3 induces cell death. Other mechanisms include opening of the permeability transition pore and the recruitment of Bax/Bak to the outer mitochondrial membrane [11]. Mitochondrial depolarization has been known for a long time to be a key step in the induction of cell death however, molecular details are unknown. Here, we suggest that sphingosine, via its biophysical interaction with cardiolipin, constitutes one of the molecules triggering mitochondrial depolarization during cell death.

Cardiolipin might be simply a binding molecule for sphingosine followed by clustering of lipids, altered membrane fluidity, and increased membrane permeabilization. It might be also possible that the binding of sphingosine to cardiolipin interferes with the function of cardiolipin in the respiratory chain. Preliminary studies suggested that the binding of sphingosine does not displace cytochrome c from mitochondria, at least early after incubation. However, cardiolipin also functions to organize proteins of the respiratory chain and thereby binding of sphingosine to cardiolipin might interfere with the organization of the respiratory chain, followed by impaired energy generation and mitochondrial depolarization.

Our data also show that the expression of oncogenic KRAS, which is one of the key oncogenes in pancreas cancer, sensitizes pancreas cancer cells to the treatment with PAPTP and promotes the formation of sphingosine in mitochondria/mitochondria-associated membranes after treatment with PAPTP. Oncogenic KRAS has been previously shown to promote the formation of reactive oxygen species in mitochondria [26]. We have previously demonstrated that the basal level of ROS production and expression levels of mitoKv1.3 determine whether these inhibitors kill cells or not [9, 11]. Since the formation of ROS upon inhibition of Kv1.3 was shown to be a crucial step in the induction of cell death [9, 11], it might be possible that oncogenic KRAS facilitates death induced by PAPTP, consistent with the present data. It is presently unknown how the formation of sphingosine is linked to the release of ROS. The acid sphingomyelinase is regulated by reactive oxygen species and redox mechanisms [29, 30]. Thus, it might be possible that reactive oxygen species also influence the activity of S1P-phosphatase.

Our in vitro and in vivo data employing ABC294640 as an inhibitor of the SK2 [33] suggests the significance of sphingosine for PAPTP-induced cell death. ABC294640 enhanced PAPTP-induced death of pancreas cancer cells in vitro and greatly delayed, in combination with PAPTP, the growth of metastatic pancreas cancer.

Kv1.3 is not only expressed in malignant cancer cells, but also in lymphocytes and macrophages. It is therefore possible that the in vivo effect observed after the application of PAPTP is not only mediated by a direct effect on malignant pancreas cancer cells, but also by killing activated immunosuppressive regulatory T lymphocytes and/or myeloid-derived suppressor cells. Such an elimination of immunosuppressive cells may then allow the immune system to become activated and contribute to the elimination of the tumor.

Our studies employed a density-based method to purify mitochondria. However, it is impossible to obtain absolutely pure mitochondria and our preparations will contain some mitochondria-associated membranes and very likely also other cellular vesicle structures. The flow cytometry studies indicate a co-localization of sphingosine with Tim23, expressed in the inner mitochondrial membrane. However, also these studies do not prove a mitochondrial formation of sphingosine and it might be possible that sphingosine is mainly formed in mitochondria-associated membranes and then transferred to mitochondria. Our studies also do not exclude that PAPTP induces the formation of sphingosine in other organelles than mitochondria (although the drug is accumulating in mitochondria by virtue of the positively charged triphenyl-phosphonium group), for instance lysosomes, and then promotes an exchange of sphingosine with mitochondria.

In summary, our data indicate that inhibition of mitochondrial Kv1.3 by PAPTP induces the formation of sphingosine, which seems to contribute to PAPTP-induced death of pancreas cancer cells in vitro and in vivo.

## Data Availability

All data are presented in the manuscript. All material is freely available.

## Abbreviations

KRAS: Kirsten Rat Sarcoma Virus
PDAC: Pancreatic ductal adenocarcinoma
S1P: Sphingosine-1 phosphate
PTP: Permeability transition pore
Gly/Asp: Glycine/aspartate
Gly/Cys: Glycine/cysteine
si-RNA: Small inhibitory RNA
Tim23: Translocase of the inner membrane 23
H/S: HEPES/saline
ROS: Reactive oxygen species
TMRM: Tetramethylrhodamine
Cy3: Cyanine 3
APC: Allophycocyanine
FITC: Fluorescein isothiocyanate
